# Penta­aqua­tri-μ_3_-hydroxido-tris­(imino­diacetato)-μ_3_-oxido-*tetra­hedro*-calcium(II)tricobalt(III) 2.54-hydrate

**DOI:** 10.1107/S1600536810010998

**Published:** 2010-03-31

**Authors:** Tomoharu Ama, Toshiaki Yonemura, Shogo Morita, Masanori Yamaguchi

**Affiliations:** a Department of Chemistry, Faculty of Science, Kochi University, Akebono-cho 2-5-1, Kochi 780-8520, Japan

## Abstract

In the title compound, [CaCo_3_(C_4_H_5_NO_4_)_3_(OH)_3_O(H_2_O)_5_]·2.54H_2_O, the Co atom is octa­hedrally coordinated by one imino­diacetate (ida) dianion as a facial *O*,*N*,*O*′-tridentate ligand, two μ_3_-OH groups and one μ_3_-O ligand, forming an partial Co_3_O_4_ cubane cluster. This unit coordinates to a Ca^II^ cation in an *O*,*O*′,*O*′′-tridentate fashion, generating a distorted CaCo_3_O_4_ cubane-type cluster. The Ca—μ_3_-O distances [2.429 (5)–2.572 (6) Å] are much longer than the Co—μ_3_—O bonds [1.895 (5)–1.941 (5) Å]. The Ca^II^ cation is also coord­inated by five water mol­ecules with Ca—O distances in the range 2.355 (6)–2.543 (6) Å. There are three additional uncoordinated water mol­ecules in the asymmetric unit, the occupancy of which refined to 0.54 (3). In H_2_O (or D_2_O), the title complex hydrolyses to Ca^2+^_aq_ cations and [Co_3_(ida)_3_(μ_2_-OH)_3_(μ_3_-O)]^2−^ anions.

## Related literature

For the synthesis and chemistry of partial Co_3_O_4_ cubane clusters, see: Ama *et al.* (1997 ▶, 2000 ▶, 2001 ▶, 2006 ▶). For the chemistry and structure of CaMn_4_O_4_ clusters in the OEC (oxygen evolution center) of plants, see: Barber & Murray (2008 ▶); Rappaport & Diner (2008 ▶); Sauer *et al.* (2008 ▶); Yocum (2008 ▶). For a related structure, see: Ama *et al.* (1995 ▶). 
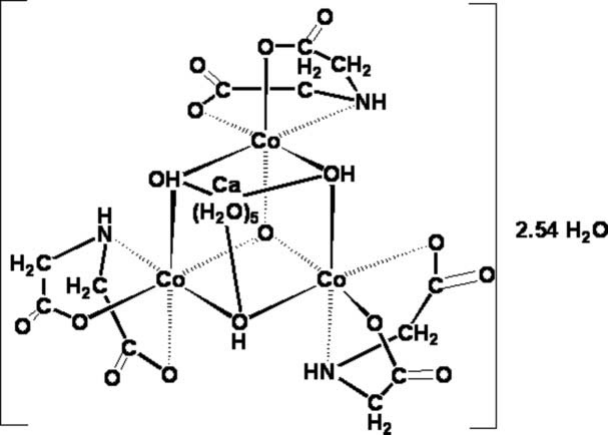


## Experimental

### 

#### Crystal data

[CaCo_3_(C_4_H_5_NO_4_)_3_(OH)_3_O(H_2_O)_5_]·2.54H_2_O
*M* *_r_* = 812.92
Triclinic, 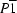

*a* = 10.474 (3) Å
*b* = 11.303 (7) Å
*c* = 12.588 (5) Å
α = 75.88 (4)°
β = 100.92 (3)°
γ = 104.58 (3)°
*V* = 1385.6 (10) Å^3^
*Z* = 2
Mo *K*α radiation
μ = 2.06 mm^−1^
*T* = 296 K
0.40 × 0.40 × 0.05 mm

#### Data collection

Rigaku AFC-7S diffractometer
Absorption correction: ψ scan (North *et al.*, 1968 ▶) *T*_min_ = 0.673, *T*_max_ = 0.902
6713 measured reflections
6360 independent reflections
3290 reflections with *F* ^2^ > 2σ(*F* ^2^)
*R*_int_ = 0.076
3 standard reflections every 150 reflections  intensity decay: 4.8%

#### Refinement

*R*[*F* ^2^ > 2σ(*F* ^2^)] = 0.060
*wR*(*F* ^2^) = 0.207
*S* = 1.00
6360 reflections
389 parameters
H-atom parameters constrained
Δρ_max_ = 1.17 e Å^−3^
Δρ_min_ = −1.13 e Å^−3^

### 

Data collection: *WinAFC* (Rigaku/MSC, 2000 ▶); cell refinement: *WinAFC*; data reduction: *CrystalStructure* (Rigaku/MSC, 2007 ▶); program(s) used to solve structure: *DIRDIF99* (Beurskens *et al.*, 1999 ▶); program(s) used to refine structure: *SHELXL97* (Sheldrick, 2008 ▶); molecular graphics: *ORTEP-3 for Windows* (Farrugia, 1997 ▶); software used to prepare material for publication: *CrystalStructure* (Rigaku/MSC, 2007 ▶).

## Supplementary Material

Crystal structure: contains datablocks global, I. DOI: 10.1107/S1600536810010998/sj2753sup1.cif

Structure factors: contains datablocks I. DOI: 10.1107/S1600536810010998/sj2753Isup2.hkl

Additional supplementary materials:  crystallographic information; 3D view; checkCIF report

## Figures and Tables

**Table 1:** Selected bond lengths (Å)

Co1—O1	1.877 (5)
Co1—O2	1.903 (5)
Co1—O4	1.941 (5)
Co2—O1	1.888 (4)
Co2—O2	1.907 (5)
Co2—O3	1.895 (5)
Co3—O1	1.866 (5)
Co3—O3	1.915 (5)
Co3—O4	1.921 (5)
Ca1—O2	2.572 (6)
Ca1—O3	2.527 (6)
Ca1—O4	2.429 (5)
Ca1—O17	2.355 (6)
Ca1—O18	2.411 (6)
Ca1—O19	2.543 (6)
Ca1—O20	2.406 (6)
Ca1—O21	2.471 (8)

**Fig. 1.**
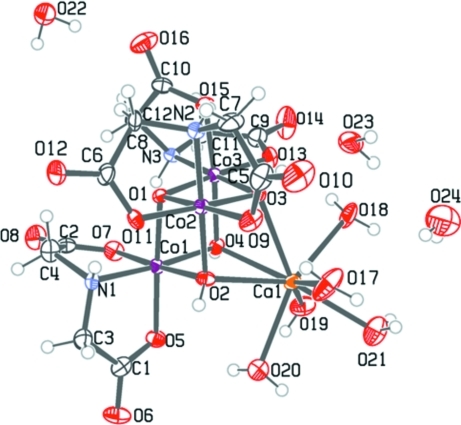
The asymmetric unit of the title compound showing the atom-labeling scheme and with 50% probability displacement ellipsoids.

## supplementary crystallographic information

### Comment

We have previously reported on the structures of some [Co_3_(L)_3_(µ_2_-OH)_3_(µ_3_-O)]^n+^ (L: tridentate ligand) complexes which form incomplete cubane Co_3_O_4_ clusters (Ama *et al.*, 1997, 2000,2001, 2006). While many such cationic [Co_3_(L)_3_(µ_2_-OH)_3_(µ_3_-O)]^n+^ complexes are known, we could find no mention of an anionic complex in our survey of the literature. The title compound was formed during an attempt to prepare the anionic complex [Co_3_(ida)_3_(µ_2_-OH)_3_(µ_3_-O)]^2-^.

Investigations of the CaMn_3_O_4_ cluster are important to gain an understanding of the chemistry of O_2_ evolution centers (OEC) in plants (Yocum, 2008; Sauer *et al.*, 2008; Barber & Murray, 2008; Rappaport & Diner, 2008). The Mn clusters in the OEC are of interest due to their unique structure, photoreaction mechanisms, and redox chemistry. Moreover, as the covalent radius of Co (1.33 Å) is similar to that of Mn (1.35 Å), we were interested in the preparation of the cobalt analogue, CaCo_3_O_4_, as a model of OEC.

In the title complex, each Co atom is coordinated by one ida (iminodiacetato) molecule which acts as a *facial* tridentate (*O,N,O'*) ligand, two µ_2_-OH groups and one µ_3_-O ligand, forming a Co_3_O_4_ cluster. This cluster further coordinates to Ca^II^ in a tridentate manner generating a distorted CaCo_3_O_4_ cubane-type cluster. The µ_2_-O—Co distances are 1.895 (5) - 1.941 (5)Å and the Co—µ_2_-O—Co' angles are 94.5 (2) – 95.8 (2)°. In the T1 isomer (Ama *et al.*, 1995) of [Co_3_(edma)_3_(µ_2_-OH)_3_(µ_3_-O)]^+^ (edma: ethylenediaminemonoacetato), the corresponding distances and angles are 1.899 (3) -1.923 (2)Å and 95.0 (1) - 96.2 (1)°. Hence the structure of the Co_3_O_4_ cluster in the title compound resembles that of [Co_3_(edma)_3_(µ_2_-OH)_3_(µ_3_-O)]^+^ cation. As the Ca^2+^ cation is coordinated by the three µ_3_-OH ligands of the Co_3_O_4_ cluster, the CaCo_3_O_4_ cluster can be considered to be a distorted cubane cluster. The Ca—µ_3_-O distances (2.429 (5) – 2.572 (6) Å) are much longer than Co—µ_3_-O (1.895 (5) - 1.921 (5) Å). The µ_3_-O—Ca—µ_3_-O' angles (59.84 (16) - 63.09 (16)) are smaller than those involving cobalt, µ_3_-O—Co—µ_3_-O' (84.0 (2) - 85.6 (2)°) and Co—µ_3_-O—Co' (94.5 (2) - 95.8 (2)) in the Co_3_O_4_ cluster. There are three additional uncoordinated water molecules in the asymmetric unit, the occupancy of one of these solvates that has the largest *U_eq_* value refines to 0.54 (3).

### Experimental

To a suspension of 7.0 g of KHCO_3_ in 10 cm^3^ of water, a solution 2.38 g of cobalt(II) chloride hexahydrate and 4 cm^3^ of 30% H_2_O_2_ in 10 cm^3^ of water was added dropwise with stirring at below 0° C. After the solution was stirred for 15 min, 10 cm^3^ of H_2_O containing 1.3 g of H_2_ida was added and then stirred overnight. The solution was acidified to pH 1.0 with 30% HClO_4_, stirred for 30 min, pH adjusted to 8.3 with 2 mol dm^-3^ KOH aqueous solution and then stirred for 2.5 h at 45°C. Filtering off the insoluble white-brown precipitate, the filtrate was loaded onto a QAE-Sephadex column (Cl^-^ form). The adsorbed band was developed with 0.2 mol dm^-3^ KCl Solution. The eluate from the fifth brown band was collected and concentrated to a small volume and then methanol was added to deposit KCl. After removing the KCl by filtration, potassium salt (K_2_[Co_3_(ida)_3_(µ_2_-OH)_3_(µ_3_-O)] 3.25 H_2_O) was obtained by standing the filtrate in a refrigerator. Yield: 76 mg. (Anal. Found: C, 18.45; H, 3.21; N, 5.34%. Calcd for C_12_H_24.5_N_3_O_19.25_K_2_Co_3_ (K_2_[Co_3_(ida)_3_(µ_2_-OH)(µ_3_-O)] 3.25 H_2_O): C, 18.62; H, 3.19; N, 5.43%. This potassium salt was dissolved in a small amount of water, which was loaded to a QAE-Sephadex column (Cl^-^ form; ø 6.5 cm × 3.5 cm) and then eluted with 500 cm^3^ of water to remove the K^+^ ion in the solution. The adsorbed brown band was eluted out with 0.5 mol dm^-3^ of CaCl_2_. The eluted solution was concentrated to a few cm^3^ and then ethanol and diethylether were added. As the solution was separated into a brown and an uncolored layers, the colorless layer was removed by decantation. After these procedures were repeated several times, a large amount of ethanol was added and the solution allowed to stand overnight at room temperature. The resulting brown precipitate was collected and washed with ethanol. This crude solid was recrystallized from water by adding ethanol. Anal. Found: C, 17.84; H, 4.04; N, 5.11%. Calcd for C_12_H_33.08_N_3_O_23.54_CaCo_3_ ([Ca(H_2_O)_5_Co_3_(ida)_3_(µ-OH)_3_(µ_3_-O)] 2.54 H_2_O): C, 17.73; H, 4.09; N, 5.17%. ^1^H NMR: (*δ*=4.22 and 3.24ppm; *J*_gem_=17.6 Hz) and (*δ*=4.03 and 3.24ppm; *J*_gem_=17.2 Hz).

### Refinement

All H atoms of the fragment containing CaCo_3_O_4_ cluster were positioned geometrically (C—H = 0.95 Å, N—H = 0.91 Å and O—H = 0.84 Å) and refined as riding, with *U*_iso_(H)= 1.2 *U*_eq_ of the parent atom. The H atoms of the solvate water molecules were found in the difference Fourier synthesis, constrained to O—H = 0.84Å and then refined as riding.

### Crystal data

**Table:**

[CaCo_3_(C_4_H_5_NO_4_)_3_(OH)_3_O(H_2_O)_5_]·2.54H_2_O	*Z* = 2
*M**_r_* = 812.92	*F*(000) = 830.80
Triclinic, *P*1	*D*_x_ = 1.948 Mg m^−^^3^
Hall symbol: -P 1	Mo *K*α radiation, λ = 0.71069 Å
*a* = 10.474 (3) Å	Cell parameters from 12 reflections
*b* = 11.303 (7) Å	θ = 15.1–15.8°
*c* = 12.588 (5) Å	µ = 2.06 mm^−^^1^
α = 75.88 (4)°	*T* = 296 K
β = 100.92 (3)°	Plate, brown
γ = 104.58 (3)°	0.40 × 0.40 × 0.05 mm
*V* = 1385.6 (10) Å^3^	

### Data collection

**Table:**

Rigaku AFC-7S diffractometer	*R*_int_ = 0.076
ω–2θ scans	θ_max_ = 27.5°
Absorption correction: ψ scan (North *et al.*, 1968)	*h* = 0→13
*T*_min_ = 0.673, *T*_max_ = 0.902	*k* = −14→14
6713 measured reflections	*l* = −16→16
6360 independent reflections	3 standard reflections every 150 reflections
3290 reflections with *F*^2^ > 2σ(*F*^2^)	intensity decay: 4.8%

### Refinement

**Table:**

Refinement on *F*^2^	H-atom parameters constrained
*R*[*F*^2^ > 2σ(*F*^2^)] = 0.060	*w* = 1/[σ^2^(*F*_o_^2^) + (0.1052*P*)^2^] where *P* = (*F*_o_^2^ + 2*F*_c_^2^)/3
*wR*(*F*^2^) = 0.207	(Δ/σ)_max_ < 0.0001
*S* = 1.00	Δρ_max_ = 1.17 e Å^−^^3^
6360 reflections	Δρ_min_ = −1.13 e Å^−^^3^
389 parameters	

### Special details

**Table:**

Geometry. ENTER SPECIAL DETAILS OF THE MOLECULAR GEOMETRY
Refinement. Refinement was performed using all reflections. The weighted *R*-factor (wR) and goodness of fit (*S*) are based on *F*^2^. *R*-factor (gt) are based on *F*. The threshold expression of *F*^2^ > 2.0 σ(*F*^2^) is used only for calculating *R*-factor (gt).

### Fractional atomic coordinates and isotropic or equivalent isotropic displacement parameters (Å^2^)

**Table:**

	*x*	*y*	*z*	*U*_iso_*/*U*_eq_	Occ. (<1)
Co(1)	0.71696 (10)	0.72533 (9)	0.93041 (8)	0.0137 (2)	
Co(2)	0.73172 (10)	0.93813 (9)	0.75658 (8)	0.0141 (2)	
Co(3)	0.51954 (10)	0.72856 (9)	0.74180 (8)	0.0140 (2)	
Ca(1)	0.81420 (15)	0.67221 (15)	0.69882 (13)	0.0190 (3)	
O(1)	0.6046 (4)	0.8286 (4)	0.8436 (4)	0.0141 (10)	
O(2)	0.8387 (5)	0.8207 (4)	0.8306 (4)	0.0176 (11)	
O(3)	0.6625 (5)	0.8240 (4)	0.6610 (4)	0.0154 (10)	
O(4)	0.6435 (5)	0.6261 (4)	0.8183 (4)	0.0160 (10)	
O(5)	0.8438 (5)	0.6279 (5)	1.0143 (4)	0.0224 (12)	
O(6)	1.0147 (6)	0.6325 (6)	1.1499 (5)	0.0377 (16)	
O(7)	0.5867 (5)	0.6299 (5)	1.0215 (4)	0.0227 (12)	
O(8)	0.4950 (6)	0.6300 (6)	1.1655 (5)	0.0327 (14)	
O(9)	0.8615 (5)	1.0388 (5)	0.6638 (4)	0.0256 (12)	
O(10)	0.8934 (7)	1.2014 (6)	0.5255 (6)	0.052 (2)	
O(11)	0.7888 (5)	1.0409 (4)	0.8634 (4)	0.0176 (11)	
O(12)	0.7064 (8)	1.1589 (7)	0.9394 (5)	0.051 (2)	
O(13)	0.4431 (5)	0.6281 (5)	0.6339 (4)	0.0228 (12)	
O(14)	0.2633 (7)	0.4911 (7)	0.5851 (6)	0.051 (2)	
O(15)	0.4032 (5)	0.8393 (5)	0.6783 (4)	0.0203 (11)	
O(16)	0.2518 (6)	0.9176 (6)	0.7259 (5)	0.0399 (16)	
O(17)	0.9506 (7)	0.8551 (6)	0.6103 (6)	0.050 (2)	
O(18)	0.6561 (6)	0.6337 (6)	0.5383 (5)	0.0358 (16)	
O(19)	0.7455 (5)	0.4350 (6)	0.7370 (5)	0.0348 (15)	
O(20)	0.9788 (6)	0.6105 (6)	0.8499 (5)	0.0313 (14)	
O(21)	0.9474 (6)	0.5947 (6)	0.5991 (5)	0.0395 (16)	
O(22)	0.0992 (5)	0.9178 (6)	0.8899 (5)	0.0315 (14)	
O(23)	0.6372 (6)	0.8910 (6)	0.4401 (5)	0.0411 (17)	
O(24)	0.8213 (16)	0.7798 (16)	0.3566 (14)	0.068 (4)	0.54 (3)
N(1)	0.7746 (6)	0.8266 (5)	1.0404 (5)	0.0168 (12)	
N(2)	0.6218 (5)	1.0576 (5)	0.6830 (5)	0.0149 (12)	
N(3)	0.3720 (6)	0.6372 (5)	0.8218 (5)	0.0167 (12)	
C(1)	0.9207 (8)	0.6772 (7)	1.0912 (6)	0.0236 (17)	
C(2)	0.5724 (7)	0.6812 (7)	1.0984 (6)	0.0195 (15)	
C(3)	0.8963 (8)	0.7956 (8)	1.1112 (7)	0.0279 (18)	
C(4)	0.6595 (8)	0.8098 (7)	1.0997 (7)	0.0251 (17)	
C(5)	0.8276 (9)	1.1327 (8)	0.5968 (7)	0.0300 (19)	
C(6)	0.7038 (9)	1.1044 (7)	0.8667 (6)	0.0253 (17)	
C(7)	0.6939 (9)	1.1555 (8)	0.6011 (7)	0.031 (2)	
C(8)	0.5893 (8)	1.1025 (7)	0.7744 (6)	0.0213 (16)	
C(9)	0.3283 (8)	0.5566 (8)	0.6488 (7)	0.0266 (18)	
C(10)	0.3213 (7)	0.8398 (7)	0.7426 (6)	0.0217 (16)	
C(11)	0.2752 (8)	0.5578 (7)	0.7508 (7)	0.0246 (17)	
C(12)	0.3138 (8)	0.7343 (7)	0.8439 (6)	0.0237 (17)	
H(1)	0.7947	0.9079	1.0048	0.020*	
H(2)	0.5452	1.0172	0.6476	0.018*	
H(3)	0.4015	0.5889	0.8864	0.020*	
H(4)	0.8859	0.7841	1.1867	0.033*	
H(5)	0.9702	0.8623	1.0944	0.033*	
H(6)	0.6091	0.8711	1.0637	0.030*	
H(7)	0.6904	0.8183	1.1738	0.030*	
H(8)	0.7057	1.2340	0.6207	0.037*	
H(9)	0.6423	1.1567	0.5303	0.037*	
H(10)	0.5117	1.0479	0.8014	0.026*	
H(11)	0.5732	1.1844	0.7478	0.026*	
H(12)	0.1962	0.5897	0.7294	0.030*	
H(13)	0.2551	0.4749	0.7923	0.030*	
H(14)	0.3632	0.7642	0.9071	0.028*	
H(15)	0.2237	0.7009	0.8558	0.028*	
H(16)	0.9166	0.8498	0.8586	0.021*	
H(17)	0.6408	0.8554	0.5940	0.018*	
H(18)	0.6107	0.5500	0.8413	0.019*	
H(19)	0.9347	0.9179	0.6258	0.060*	
H(20)	1.0115	0.8682	0.5710	0.060*	
H(21)	0.6803	0.5862	0.5078	0.043*	
H(22)	0.5806	0.5997	0.5577	0.043*	
H(23)	0.7839	0.4056	0.7991	0.042*	
H(24)	0.6626	0.4130	0.7365	0.042*	
H(25)	0.9410	0.5690	0.9048	0.038*	
H(26)	1.0209	0.5666	0.8310	0.038*	
H(27)	0.8977	0.5387	0.5677	0.047*	
H(28)	0.9823	0.6540	0.5514	0.047*	
H(29)	0.1383	0.8707	0.9402	0.038*	
H(30)	0.1340	0.8916	0.8478	0.038*	
H(31)	0.6683	0.8379	0.4229	0.049*	
H(32)	0.6765	0.9500	0.3952	0.049*	
H(33)	0.8162	0.7109	0.3878	0.079*	0.54
H(34)	0.8708	0.8215	0.3978	0.079*	0.54

### Atomic displacement parameters (Å^2^)

**Table:**

	*U*^11^	*U*^22^	*U*^33^	*U*^12^	*U*^13^	*U*^23^
Co(1)	0.0127 (4)	0.0144 (5)	0.0121 (4)	0.0010 (3)	0.0026 (3)	−0.0006 (3)
Co(2)	0.0116 (4)	0.0139 (5)	0.0152 (4)	0.0011 (3)	0.0034 (3)	−0.0007 (3)
Co(3)	0.0110 (4)	0.0161 (5)	0.0135 (4)	0.0006 (3)	0.0026 (3)	−0.0024 (3)
Ca(1)	0.0147 (7)	0.0214 (8)	0.0208 (7)	0.0042 (6)	0.0039 (6)	−0.0027 (6)
O(1)	0.012 (2)	0.014 (2)	0.018 (2)	0.003 (2)	−0.0010 (19)	−0.008 (2)
O(2)	0.013 (2)	0.016 (2)	0.020 (2)	0.002 (2)	0.000 (2)	−0.001 (2)
O(3)	0.018 (2)	0.015 (2)	0.013 (2)	0.003 (2)	0.004 (2)	−0.001 (2)
O(4)	0.018 (2)	0.012 (2)	0.018 (2)	0.004 (2)	0.005 (2)	−0.003 (2)
O(5)	0.025 (2)	0.018 (2)	0.023 (2)	0.008 (2)	0.001 (2)	0.000 (2)
O(6)	0.032 (3)	0.039 (3)	0.038 (3)	0.019 (3)	−0.007 (2)	0.001 (3)
O(7)	0.020 (2)	0.018 (2)	0.024 (2)	−0.004 (2)	0.007 (2)	0.001 (2)
O(8)	0.029 (3)	0.034 (3)	0.032 (3)	−0.003 (2)	0.017 (2)	−0.002 (2)
O(9)	0.020 (2)	0.021 (2)	0.034 (3)	0.001 (2)	0.015 (2)	0.002 (2)
O(10)	0.058 (4)	0.040 (4)	0.057 (4)	0.018 (3)	0.039 (4)	0.025 (3)
O(11)	0.020 (2)	0.017 (2)	0.015 (2)	0.000 (2)	−0.002 (2)	−0.009 (2)
O(12)	0.067 (5)	0.073 (5)	0.033 (3)	0.046 (4)	−0.014 (3)	−0.033 (3)
O(13)	0.015 (2)	0.031 (3)	0.019 (2)	−0.007 (2)	0.009 (2)	−0.006 (2)
O(14)	0.035 (3)	0.069 (5)	0.052 (4)	−0.022 (3)	0.012 (3)	−0.045 (4)
O(15)	0.016 (2)	0.023 (2)	0.021 (2)	0.007 (2)	0.002 (2)	−0.001 (2)
O(16)	0.034 (3)	0.038 (3)	0.050 (4)	0.019 (3)	0.020 (3)	0.008 (3)
O(17)	0.041 (4)	0.034 (3)	0.083 (5)	0.003 (3)	0.043 (4)	−0.005 (3)
O(18)	0.028 (3)	0.048 (4)	0.045 (3)	0.006 (3)	0.010 (2)	−0.033 (3)
O(19)	0.020 (3)	0.033 (3)	0.047 (3)	0.002 (2)	0.006 (2)	−0.002 (3)
O(20)	0.030 (3)	0.036 (3)	0.030 (3)	0.009 (2)	0.004 (2)	−0.009 (2)
O(21)	0.040 (3)	0.029 (3)	0.051 (4)	−0.003 (2)	0.025 (3)	−0.007 (3)
O(22)	0.020 (2)	0.039 (3)	0.037 (3)	0.002 (2)	0.002 (2)	−0.016 (2)
O(23)	0.039 (3)	0.053 (4)	0.029 (3)	0.020 (3)	0.009 (3)	0.010 (3)
O(24)	0.063 (10)	0.071 (11)	0.066 (10)	0.016 (9)	0.003 (8)	−0.009 (9)
N(1)	0.021 (3)	0.015 (3)	0.015 (3)	0.003 (2)	0.006 (2)	−0.004 (2)
N(2)	0.013 (2)	0.013 (2)	0.017 (3)	−0.001 (2)	0.003 (2)	−0.001 (2)
N(3)	0.015 (3)	0.017 (3)	0.016 (3)	0.004 (2)	0.000 (2)	−0.001 (2)
C(1)	0.023 (4)	0.018 (4)	0.029 (4)	0.004 (3)	0.010 (3)	0.001 (3)
C(2)	0.015 (3)	0.019 (3)	0.023 (3)	0.003 (2)	0.001 (3)	−0.002 (3)
C(3)	0.026 (4)	0.034 (4)	0.028 (4)	0.008 (3)	0.002 (3)	−0.014 (3)
C(4)	0.024 (4)	0.022 (4)	0.027 (4)	−0.004 (3)	0.009 (3)	−0.006 (3)
C(5)	0.028 (4)	0.025 (4)	0.037 (5)	−0.002 (3)	0.014 (3)	−0.008 (3)
C(6)	0.038 (4)	0.021 (4)	0.023 (4)	0.014 (3)	0.005 (3)	−0.008 (3)
C(7)	0.036 (4)	0.023 (4)	0.034 (4)	0.018 (3)	0.012 (4)	0.013 (3)
C(8)	0.029 (4)	0.025 (4)	0.015 (3)	0.010 (3)	0.005 (3)	−0.007 (3)
C(9)	0.019 (3)	0.029 (4)	0.031 (4)	0.000 (3)	0.005 (3)	−0.009 (3)
C(10)	0.016 (3)	0.021 (4)	0.025 (4)	0.003 (3)	−0.001 (3)	−0.003 (3)
C(11)	0.019 (3)	0.023 (4)	0.035 (4)	−0.005 (3)	0.009 (3)	−0.016 (3)
C(12)	0.022 (4)	0.022 (4)	0.027 (4)	0.004 (3)	0.011 (3)	0.000 (3)

### Geometric parameters (Å, °)

**Table:**

Co(1)—O(1)	1.877 (5)	N(3)—C(11)	1.491 (10)
Co(1)—O(2)	1.903 (5)	N(3)—C(12)	1.484 (12)
Co(1)—O(4)	1.941 (5)	C(1)—C(3)	1.511 (14)
Co(1)—O(5)	1.932 (5)	C(2)—C(4)	1.511 (10)
Co(1)—O(7)	1.894 (5)	C(5)—C(7)	1.499 (14)
Co(1)—N(1)	1.923 (6)	C(6)—C(8)	1.503 (11)
Co(2)—O(1)	1.888 (4)	C(9)—C(11)	1.498 (14)
Co(2)—O(2)	1.907 (5)	C(10)—C(12)	1.518 (10)
Co(2)—O(3)	1.895 (5)	O(2)—H(16)	0.842
Co(2)—O(9)	1.918 (5)	O(3)—H(17)	0.843
Co(2)—O(11)	1.904 (5)	O(4)—H(18)	0.842
Co(2)—N(2)	1.943 (6)	O(17)—H(19)	0.841
Co(3)—Ca(1)	3.466 (2)	O(17)—H(20)	0.844
Co(3)—O(1)	1.866 (5)	O(18)—H(21)	0.840
Co(3)—O(3)	1.915 (5)	O(18)—H(22)	0.840
Co(3)—O(4)	1.921 (5)	O(19)—H(23)	0.840
Co(3)—O(13)	1.913 (6)	O(19)—H(24)	0.839
Co(3)—O(15)	1.897 (5)	O(20)—H(25)	0.840
Co(3)—N(3)	1.936 (6)	O(20)—H(26)	0.840
Ca(1)—O(2)	2.572 (6)	O(21)—H(27)	0.840
Ca(1)—O(3)	2.527 (6)	O(21)—H(28)	0.840
Ca(1)—O(4)	2.429 (5)	O(22)—H(29)	0.837
Ca(1)—O(17)	2.355 (6)	O(22)—H(30)	0.839
Ca(1)—O(18)	2.411 (6)	O(23)—H(31)	0.837
Ca(1)—O(19)	2.543 (6)	O(23)—H(32)	0.836
Ca(1)—O(20)	2.406 (6)	O(24)—H(33)	0.775
Ca(1)—O(21)	2.471 (8)	O(24)—H(34)	0.809
O(5)—C(1)	1.280 (9)	N(1)—H(1)	0.910
O(6)—C(1)	1.249 (10)	N(2)—H(2)	0.910
O(7)—C(2)	1.292 (11)	N(3)—H(3)	0.910
O(8)—C(2)	1.220 (10)	C(3)—H(4)	0.951
O(9)—C(5)	1.267 (10)	C(3)—H(5)	0.951
O(10)—C(5)	1.227 (11)	C(4)—H(6)	0.951
O(11)—C(6)	1.288 (12)	C(4)—H(7)	0.947
O(12)—C(6)	1.215 (13)	C(7)—H(8)	0.951
O(13)—C(9)	1.287 (9)	C(7)—H(9)	0.950
O(14)—C(9)	1.219 (12)	C(8)—H(10)	0.952
O(15)—C(10)	1.288 (11)	C(8)—H(11)	0.950
O(16)—C(10)	1.234 (12)	C(11)—H(12)	0.951
N(1)—C(3)	1.477 (10)	C(11)—H(13)	0.948
N(1)—C(4)	1.485 (12)	C(12)—H(14)	0.951
N(2)—C(7)	1.475 (10)	C(12)—H(15)	0.951
N(2)—C(8)	1.489 (12)		
			
O(1)—Co(1)—O(2)	83.4 (2)	Co(2)—O(11)—C(6)	113.5 (4)
O(1)—Co(1)—O(4)	82.8 (2)	Co(3)—O(13)—C(9)	115.3 (6)
O(1)—Co(1)—O(5)	175.6 (2)	Co(3)—O(15)—C(10)	113.6 (4)
O(1)—Co(1)—O(7)	93.8 (2)	Co(1)—N(1)—C(3)	110.3 (6)
O(1)—Co(1)—N(1)	93.2 (2)	Co(1)—N(1)—C(4)	107.1 (4)
O(2)—Co(1)—O(4)	85.6 (2)	C(3)—N(1)—C(4)	115.2 (6)
O(2)—Co(1)—O(5)	92.3 (2)	Co(2)—N(2)—C(7)	109.6 (5)
O(2)—Co(1)—O(7)	176.1 (2)	Co(2)—N(2)—C(8)	104.6 (4)
O(2)—Co(1)—N(1)	97.0 (2)	C(7)—N(2)—C(8)	115.1 (6)
O(4)—Co(1)—O(5)	97.9 (2)	Co(3)—N(3)—C(11)	108.5 (5)
O(4)—Co(1)—O(7)	91.4 (2)	Co(3)—N(3)—C(12)	105.2 (4)
O(4)—Co(1)—N(1)	175.0 (2)	C(11)—N(3)—C(12)	111.8 (6)
O(5)—Co(1)—O(7)	90.5 (2)	O(5)—C(1)—O(6)	123.4 (8)
O(5)—Co(1)—N(1)	86.3 (2)	O(5)—C(1)—C(3)	117.9 (7)
O(7)—Co(1)—N(1)	85.8 (2)	O(6)—C(1)—C(3)	118.7 (7)
O(1)—Co(2)—O(2)	82.9 (2)	O(7)—C(2)—O(8)	122.9 (7)
O(1)—Co(2)—O(3)	82.7 (2)	O(7)—C(2)—C(4)	114.9 (6)
O(1)—Co(2)—O(9)	175.7 (2)	O(8)—C(2)—C(4)	122.2 (8)
O(1)—Co(2)—O(11)	91.0 (2)	N(1)—C(3)—C(1)	110.2 (7)
O(1)—Co(2)—N(2)	96.5 (2)	N(1)—C(4)—C(2)	109.2 (7)
O(2)—Co(2)—O(3)	84.0 (2)	O(9)—C(5)—O(10)	124.4 (9)
O(2)—Co(2)—O(9)	94.7 (2)	O(9)—C(5)—C(7)	117.0 (7)
O(2)—Co(2)—O(11)	94.9 (2)	O(10)—C(5)—C(7)	118.4 (8)
O(2)—Co(2)—N(2)	179.2 (2)	O(11)—C(6)—O(12)	125.5 (8)
O(3)—Co(2)—O(9)	93.4 (2)	O(11)—C(6)—C(8)	115.3 (8)
O(3)—Co(2)—O(11)	173.8 (2)	O(12)—C(6)—C(8)	119.1 (9)
O(3)—Co(2)—N(2)	96.4 (2)	N(2)—C(7)—C(5)	111.7 (7)
O(9)—Co(2)—O(11)	92.8 (2)	N(2)—C(8)—C(6)	109.6 (7)
O(9)—Co(2)—N(2)	86.0 (2)	O(13)—C(9)—O(14)	123.7 (9)
O(11)—Co(2)—N(2)	84.6 (2)	O(13)—C(9)—C(11)	116.2 (7)
Ca(1)—Co(3)—O(1)	93.60 (17)	O(14)—C(9)—C(11)	120.2 (7)
Ca(1)—Co(3)—O(3)	45.55 (17)	O(15)—C(10)—O(16)	124.6 (7)
Ca(1)—Co(3)—O(4)	42.59 (16)	O(15)—C(10)—C(12)	114.8 (7)
Ca(1)—Co(3)—O(13)	82.93 (19)	O(16)—C(10)—C(12)	120.6 (8)
Ca(1)—Co(3)—O(15)	139.35 (16)	N(3)—C(11)—C(9)	112.4 (6)
Ca(1)—Co(3)—N(3)	136.5 (2)	N(3)—C(12)—C(10)	107.0 (7)
O(1)—Co(3)—O(3)	82.8 (2)	Co(1)—O(2)—H(16)	117.0
O(1)—Co(3)—O(4)	83.6 (2)	Co(2)—O(2)—H(16)	116.9
O(1)—Co(3)—O(13)	176.3 (2)	Ca(1)—O(2)—H(16)	117.0
O(1)—Co(3)—O(15)	90.4 (2)	Co(2)—O(3)—H(17)	116.3
O(1)—Co(3)—N(3)	96.7 (2)	Co(3)—O(3)—H(17)	116.2
O(3)—Co(3)—O(4)	85.1 (2)	Ca(1)—O(3)—H(17)	116.2
O(3)—Co(3)—O(13)	93.8 (2)	Co(1)—O(4)—H(18)	116.3
O(3)—Co(3)—O(15)	95.2 (2)	Co(3)—O(4)—H(18)	116.3
O(3)—Co(3)—N(3)	177.9 (2)	Ca(1)—O(4)—H(18)	116.4
O(4)—Co(3)—O(13)	94.6 (2)	Ca(1)—O(17)—H(19)	109.5
O(4)—Co(3)—O(15)	174.0 (2)	Ca(1)—O(17)—H(20)	133.3
O(4)—Co(3)—N(3)	96.9 (2)	H(19)—O(17)—H(20)	117.1
O(13)—Co(3)—O(15)	91.3 (2)	Ca(1)—O(18)—H(21)	109.5
O(13)—Co(3)—N(3)	86.8 (2)	Ca(1)—O(18)—H(22)	109.5
O(15)—Co(3)—N(3)	82.8 (2)	H(21)—O(18)—H(22)	109.4
Co(3)—Ca(1)—O(2)	64.34 (12)	Ca(1)—O(19)—H(23)	109.4
Co(3)—Ca(1)—O(3)	32.76 (10)	Ca(1)—O(19)—H(24)	109.4
Co(3)—Ca(1)—O(4)	32.37 (12)	H(23)—O(19)—H(24)	109.6
Co(3)—Ca(1)—O(17)	111.7 (2)	Ca(1)—O(20)—H(25)	109.5
Co(3)—Ca(1)—O(18)	74.12 (19)	Ca(1)—O(20)—H(26)	109.5
Co(3)—Ca(1)—O(19)	97.28 (16)	H(25)—O(20)—H(26)	109.5
Co(3)—Ca(1)—O(20)	121.95 (18)	Ca(1)—O(21)—H(27)	109.4
Co(3)—Ca(1)—O(21)	153.06 (16)	Ca(1)—O(21)—H(28)	109.4
O(2)—Ca(1)—O(3)	59.84 (16)	H(27)—O(21)—H(28)	109.5
O(2)—Ca(1)—O(4)	62.91 (17)	H(29)—O(22)—H(30)	84.2
O(2)—Ca(1)—O(17)	78.2 (2)	H(31)—O(23)—H(32)	93.0
O(2)—Ca(1)—O(18)	132.2 (2)	H(33)—O(24)—H(34)	105.7
O(2)—Ca(1)—O(19)	128.8 (2)	Co(1)—N(1)—H(1)	108.0
O(2)—Ca(1)—O(20)	75.0 (2)	C(3)—N(1)—H(1)	108.1
O(2)—Ca(1)—O(21)	141.50 (19)	C(4)—N(1)—H(1)	107.9
O(3)—Ca(1)—O(4)	63.09 (16)	Co(2)—N(2)—H(2)	109.1
O(3)—Ca(1)—O(17)	79.3 (2)	C(7)—N(2)—H(2)	109.1
O(3)—Ca(1)—O(18)	72.5 (2)	C(8)—N(2)—H(2)	109.2
O(3)—Ca(1)—O(19)	126.86 (18)	Co(3)—N(3)—H(3)	110.4
O(3)—Ca(1)—O(20)	134.5 (2)	C(11)—N(3)—H(3)	110.3
O(3)—Ca(1)—O(21)	139.57 (19)	C(12)—N(3)—H(3)	110.5
O(4)—Ca(1)—O(17)	135.4 (2)	N(1)—C(3)—H(4)	109.3
O(4)—Ca(1)—O(18)	93.7 (2)	N(1)—C(3)—H(5)	109.3
O(4)—Ca(1)—O(19)	76.6 (2)	C(1)—C(3)—H(4)	109.3
O(4)—Ca(1)—O(20)	92.9 (2)	C(1)—C(3)—H(5)	109.3
O(4)—Ca(1)—O(21)	148.6 (2)	H(4)—C(3)—H(5)	109.4
O(17)—Ca(1)—O(18)	97.1 (2)	N(1)—C(4)—H(6)	109.5
O(17)—Ca(1)—O(19)	148.0 (2)	N(1)—C(4)—H(7)	109.7
O(17)—Ca(1)—O(20)	97.6 (2)	C(2)—C(4)—H(6)	109.3
O(17)—Ca(1)—O(21)	76.0 (2)	C(2)—C(4)—H(7)	109.5
O(18)—Ca(1)—O(19)	77.4 (2)	H(6)—C(4)—H(7)	109.6
O(18)—Ca(1)—O(20)	151.5 (2)	N(2)—C(7)—H(8)	108.7
O(18)—Ca(1)—O(21)	79.4 (2)	N(2)—C(7)—H(9)	108.8
O(19)—Ca(1)—O(20)	77.2 (2)	C(5)—C(7)—H(8)	109.0
O(19)—Ca(1)—O(21)	72.0 (2)	C(5)—C(7)—H(9)	109.1
O(20)—Ca(1)—O(21)	80.6 (2)	H(8)—C(7)—H(9)	109.4
Co(1)—O(1)—Co(2)	97.1 (2)	N(2)—C(8)—H(10)	109.5
Co(1)—O(1)—Co(3)	98.6 (2)	N(2)—C(8)—H(11)	109.6
Co(2)—O(1)—Co(3)	97.7 (2)	C(6)—C(8)—H(10)	109.4
Co(1)—O(2)—Co(2)	95.6 (2)	C(6)—C(8)—H(11)	109.5
Co(1)—O(2)—Ca(1)	101.5 (2)	H(10)—C(8)—H(11)	109.3
Co(2)—O(2)—Ca(1)	105.8 (2)	N(3)—C(11)—H(12)	108.6
Co(2)—O(3)—Co(3)	95.8 (2)	N(3)—C(11)—H(13)	108.8
Co(2)—O(3)—Ca(1)	107.9 (2)	C(9)—C(11)—H(12)	108.7
Co(3)—O(3)—Ca(1)	101.7 (2)	C(9)—C(11)—H(13)	108.8
Co(1)—O(4)—Co(3)	94.5 (2)	H(12)—C(11)—H(13)	109.6
Co(1)—O(4)—Ca(1)	105.5 (2)	N(3)—C(12)—H(14)	110.2
Co(3)—O(4)—Ca(1)	105.0 (2)	N(3)—C(12)—H(15)	110.3
Co(1)—O(5)—C(1)	113.7 (5)	C(10)—C(12)—H(14)	110.0
Co(1)—O(7)—C(2)	115.1 (4)	C(10)—C(12)—H(15)	110.0
Co(2)—O(9)—C(5)	115.6 (6)	H(14)—C(12)—H(15)	109.4

### Hydrogen-bond geometry (Å, °)

**Table:**

*D*—H···*A*	*D*—H	H···*A*	*D*···*A*	*D*—H···*A*
O(2)—H(16)···O(22)^i^	0.84	1.88	2.707 (7)	168
O(3)—H(17)···O(23)	0.84	1.88	2.677 (7)	159
O(4)—H(18)···O(8)^ii^	0.84	2.07	2.870 (7)	158
O(17)—H(19)···O(9)	0.84	1.92	2.744 (11)	168
O(17)—H(20)···O(10)^iii^	0.84	2.10	2.850 (13)	148
O(18)—H(21)···O(14)^iv^	0.84	1.87	2.699 (12)	168
O(18)—H(22)···O(13)	0.84	2.00	2.708 (9)	141
O(19)—H(23)···O(6)^v^	0.84	2.21	2.829 (9)	131
O(19)—H(24)···O(8)^ii^	0.84	2.14	2.863 (9)	145
O(20)—H(25)···O(5)	0.84	2.17	2.787 (9)	130
O(20)—H(26)···O(6)^v^	0.84	2.15	2.765 (10)	130
O(21)—H(27)···O(14)^iv^	0.84	2.34	3.043 (9)	142
O(21)—H(28)···O(10)^iii^	0.84	1.99	2.801 (9)	164
O(22)—H(29)···O(12)^vi^	0.84	2.04	2.800 (9)	152
O(22)—H(30)···O(16)	0.84	2.07	2.840 (10)	153
O(23)—H(31)···O(18)	0.84	2.40	2.909 (10)	120
O(23)—H(31)···O(24)	0.84	2.24	3.01 (2)	153
O(23)—H(32)···O(16)^vii^	0.84	1.97	2.802 (8)	172
O(24)—H(33)···O(14)^iv^	0.78	2.19	2.912 (18)	156
O(24)—H(34)···O(10)^iii^	0.81	2.53	3.054 (18)	124
N(1)—H(1)···O(11)	0.91	2.03	2.857 (7)	150
N(1)—H(1)···O(22)^vi^	0.91	2.55	3.121 (9)	122
N(2)—H(2)···O(15)	0.91	2.18	2.917 (7)	138
N(2)—H(2)···O(23)^vii^	0.91	2.36	2.977 (9)	125
N(3)—H(3)···O(7)	0.91	2.36	3.038 (7)	132
N(3)—H(3)···O(7)^ii^	0.91	2.49	3.259 (8)	143

Symmetry codes: (i) *x*+1, *y*, *z*; (ii) −*x*+1, −*y*+1, −*z*+2; (iii) −*x*+2, −*y*+2, −*z*+1; (iv) −*x*+1, −*y*+1, −*z*+1; (v) −*x*+2, −*y*+1, −*z*+2; (vi) −*x*+1, −*y*+2, −*z*+2; (vii) −*x*+1, −*y*+2, −*z*+1.
